# Developing a student-led health and wellbeing clinic in an underserved community: collaborative learning, health outcomes and cost savings

**DOI:** 10.1186/s12912-015-0083-9

**Published:** 2015-05-14

**Authors:** Cynthia M. Stuhlmiller, Barry Tolchard

**Affiliations:** School of Health, University of New England, Armidale, NSW 2351 Australia

**Keywords:** Rural health, Primary health care, Student-led clinic, Aboriginal health

## Abstract

**Background:**

The University of New England (UNE), Australia decided to develop innovative placement opportunities for its increasing numbers of nursing students. Extensive community and stakeholder consultation determined that a community centre in rural New South Wales was the welcomed site of the student-led clinic because it fit the goals of the project—to increase access to health care services in an underserved area while providing service learning for students.

**Methods:**

Supported by a grant from Health Workforce Australia and in partnership with several community organisations, UNE established a student-led clinic in a disadvantaged community using an engaged scholarship approach which joins academic service learning with community based action research. The clinic was managed and run by the students, who were supervised by university staff and worked in collaboration with residents and local health and community services.

**Results:**

Local families, many of whom were Indigenous Australians, received increased access to culturally appropriate health services. In the first year, the clinic increased from a one day per week to a three day per week service and offered over 1000 occasions of care and involved 1500 additional community members in health promotion activities. This has led to improved health outcomes for the community and cost savings to the health service estimated to be $430,000. The students learned from members of the community and community members learned from the students, in a collaborative process. Community members benefited from access to drop in help that was self-determined.

**Conclusions:**

The model of developing student-led community health and wellbeing clinics in underserved communities not only fulfils the local, State Government, Federal Government and international health reform agenda but it also represents good value for money. It offers free health services in a disadvantaged community, thereby improving overall health and wellbeing. The student-led clinic is an invaluable and sustainable link between students, health care professionals, community based organisations, the university, and the community. The community benefits from the clinic by learning to self-manage health and wellbeing issues. The benefits for students are that they gain practical experience in an interdisciplinary setting and through exposure to a community with unique and severe needs.

## Background

In this section we will briefly discuss the shortages and difficulties with access to health services in a disadvantaged area of rural NSW, the benefits of student-led clinics and the reasons for this study.

### Shortages and difficulties with access to health care services

Shortages in the health workforce and distribution of the health care resources contribute to health inequities. Health professional shortages are associated with poorer general health status, worse physical health and less access to health care, thus creating a vicious cycle [1].

For Indigenous populations, poor access to culturally appropriate primary health services is believed to have contributed to poor outcomes for a wide range of chronic diseases, many of which are fully preventable [2–5]. The major health concerns for Indigenous people include diabetes [6], smoking-related cancers [3, 7], cardiovascular disease, respiratory ailments such as asthma, trachoma, and mental health issues [8]. These health issues are compounded by alcohol or drug abuse, domestic violence, falls and other injuries, and the distress experienced when a family member dies or is sent to prison [9, 10].

Australia’s Indigenous people have even poorer access to primary health services and are more likely to need hospital treatment than non-Indigenous people [11]. When it comes to mortality rates, figures from the Australian Bureau of Statistics (2010–2012) show a ten year disparity between life expectancy for Indigenous and non-Indigenous Australians. An Indigenous male is likely to live to 69.1 years compared to a non-Indigenous man, who could expect to live to 79.7 years. An Indigenous woman is likely to live to 73.7 years compared to a non-Indigenous woman, who could expect to live 83.2 years [12].

While these statistics are grim, research worldwide indicates that the biggest health gains for Indigenous people come from preventative approaches and management of chronic disease in a primary health care setting [13]. Successful models involve consumer-driven Indigenous-delivered services which have shown to improve access and health status. As with all consumer-focused models, the person, family and community are in the centre of the system and determine and control the method and type of service [14]. One health delivery model that embraces a community driven client focused approach is the student-led clinic.

### The benefits of student-led clinics

National and international shortages of clinical placements for nursing students has been documented for some time [15]. The reasons identified for the supply not meeting the demands include insufficient space, lack of equipment, lack of supervisors, competition between universities, reduced bed numbers, higher patient throughput, staff shortages, increased staff workload, the perceived burden associated with precepting students, and the limited ability of a clinical agency to accept increased numbers of students [15–17].

One solution to the shortage of placements has been the development of student-led clinics. Student-delivered, student-run, student-assisted health services are part of a long and strong tradition worldwide [18–30]. The Society for Student-Run Free Clinics reports over 147 registered student clinics. Student-led clinics are numerous in North America with over 100 currently in operation serving 36,000 patients annually [31].

The models and approaches adopted by student-led clinics vary widely and range from: a) inpatient to community-based, b) general to disease-specific, c) inter-professional to one discipline, d) large centres to small clinics, e) funded to unfunded services, f) all volunteer students to mix of students and professionals g) paid and unpaid, f) short to long term involvement, and g) public patients to private patients. However, the common thread is that they provide students with greater learning through taking charge of the management and delivery of a health service during clinical training.

Additional reported benefits for student learning include increased communication, collaboration and leadership skills [21–23, 32]. From the client perspective, student-led clinics can enable increased access to services, more time for assessments and treatments, increased depth of health teaching, more holistic and integrated care, and in most cases free help and support.

### Reasons for this study

The significance of this project was twofold: 1) it focused on a wide range of health priority areas and 2) increased student learning and leadership capacity. The project was strategically aligned with local and national health care agendas including the Australian Health Care Reform Alliance, Department of Health and Aging, and New South Wales Health namely by: a) increasing access to integrated help, b) providing efficient treatments that work in a primary health setting, c) promoting individuals to optimize their own health, d) increasing health literacy and community engagement, e) initiating new ways of working toward workforce development, f) use of e-health, g) addressing key areas of chronic and preventable disease self-management and mental health, and h) liaising with Medicare Locals (primary care providers) to fill the gap in primary care needs of rural populations. Furthermore, the location of the clinic in an underserved community provided the double advantage of improved health outcomes for people living with disadvantage in rural NSW and clinical placements and opportunities for collaborative learning for nursing students at UNE. This study was conducted to evaluate the outcomes of the development of this student-led clinic.

## Methods

In 2012, Health Workforce Australia (HWA) awarded a grant to the UNE Nursing Program in Australia, to facilitate the establishment of a student-led clinic as a means to address the shortage of nursing clinical training opportunities. While the main aim of the grant was to increase nursing enrollments at the university by increasing capacity for clinical education, the ability to offer student-delivered health services to a community with little or no access to health help created synergistic outcomes for both students and community members. In this methods section, we will discuss the aims of the clinic, the community context for the clinic, the research approach and process involved in setting up the student-led clinic, the 4G approach used, and the requirement for record keeping.

### Aims and objectives of the clinic

The main aim of the clinic was to build health literacy, equity and access to health help in an underserved community through providing learning opportunities that were community driven, student led, person-centered, evolving, sustainable and inter-professional. For students to meet this aim they were to:

1. effectively engage with the community, its leaders and other stakeholders in assessing and responding to health and social well-being needs,
2. increase provision of evidence-based integrated health help that promotes collaborative learning, self-determination and responsibility, and
3. demonstrate positive health outcomes as determined by standardized measures.

### The community context for the clinic

The Coledale Community Centre in West Tamworth, New South Wales, Australia was selected as the clinic site because of its fit with the philosophy and goals of the project. Coledale was a community of over 3000 residents with high rates of unemployment, domestic violence, crime and unmet health needs [33]. There was a high unemployment rate of almost triple the Australian base level; a high proportion of people employed as labourers (double the Australian base level) and lower number of people employed as professionals (60 % less than the Australian base level); a significantly lower number of couple families with children (30 % less) while the number of one parent families was double the Australian base level; and a significantly lower level of education and qualification (with almost double the Australian base level of people with no formal qualifications). A Crime Review in late 2011 indicated that Coledale has had a significant impact on crime rates in the Tamworth Sector [34].

The proportion of Indigenous Australians living in Coledale was much higher than in Australia overall, with 28.8 % identifying as Aboriginal and Torres Strait Islander (ATSI) in the last census of 2006 [34]. However, the Indigenous population was believed to be much higher—nearly 70 %. There was also a severe shortage of GPs in the Tamworth region, with over 2000 patients per full-time GP compared with the NSW State average of 1133 [35]. Given the lack of GPs, this meant that many Indigenous people were left without access to suitable health care services.

In mid-2010, a Coledale Action Plan was prepared as the basis for a whole of government response to address these community conditions. Identified in the plan were strategies relating to urban revitalization, antisocial behaviours and crime prevention initiatives, improving access to support services, developing youth opportunities, improving education and employment outcome, and health and well-being [33].

### The process involved in setting up the student-led clinic

In 2012, the principal investigator attended several Coledale Action Plan meetings with local service providers including training, employment, education, housing, social services, police and health agencies. Many of these groups were operating from the Coledale Community Centre—the only public space in the community. Despite the wide range of programs and initiatives held at the Community Centre, attempts to break the cycle of generational social disadvantage had been unsuccessful. At the same time, attendance at the Community Centre had been dropping, vandalism was on the increase, and staff members were feeling unsafe. The principal investigator saw this as an opportunity to bring a student workforce who could dedicate themselves to working across agencies to address the health and well-being of this community within the context of current activity at the Community Centre, while meeting student learning needs.

The principle investigator, skilled in engaged scholarship research, applied these techniques to the development and study of the project. Engaged scholarship is defined as scholarship that puts the academic resources of the university to work in solving public problems. Engaged scholarship joins academic service learning with community action research. Academic service learning is about developing and translating academic knowledge while community based action research responds to community-identified needs through civic engagement [36]. The three major principles of community participation action research were applied in the development of the clinic, namely: 1) commitment to social transformation, 2) honouring the lived experiences and knowledge of participants, and 3) collaboration and power sharing [37]. Deliberate strategies of publically engaged scholarship defined the role of the academic researchers to acknowledge and embrace their involvement in the everyday politics of the community centring on the relations and interactions of people involved in the work of the community [38]. Publically engaged scholarship focused all activity as an intellectual endeavour jointly planned and carried out by the university and community with an aim to produce public good [39].

As such, academics worked in reciprocal partnership with the community and interdisciplinary agencies, while integrating their faculty roles of teaching, research and service. Thus, individuals from within and outside of the academy joined to seek mutually beneficial exchange of knowledge and resources to solve the access to health issues of the Coledale community in ways to “advanced the public good with and not merely for the public” [40].

A year of consultation with key stakeholders and agencies occurred between 2012 and 2013 with letters of support for the development of a student-led clinic. An 8-min video of a similar clinic in North American helped to provide an understanding of what was being proposed. A steering committee composed of community members, elders, agency leads and students convened to inform and evaluate each step of actions taken in the development of the clinic. Ongoing feedback and recommendations from the committee provided invaluable direction as the form and shape of the clinic evolved. A strategic plan was written that included mission, goals, governance structure, risk management strategy, research and education committee, and budget. This plan not only provided a clear framework and timeline for program development it helped track the milestones and identify areas of concern that were brought to the steering committee for problem solving. Students developed the day-to-day operation of the clinic, advertisement and marketing materials and student orientation booklet. A website was set up to include media reports, calendar of events and discussion forum for students and community members. As a member of both the Coledale Action Plan Committee and the Coledale Neighbourhood Opportunities Working Committee, the lead investigator regularly reported progress to these groups and obtained additional feedback and support. All minutes of meetings were supplied to committee members and available to anyone interested to enable transparency and further collaborative input.

The grant funding enabled UNE to renovate the Community Centre premises in exchange for a 10-year lease on parts of the building for student education. The renovations were undertaken in accordance with practice standards, to enable practitioners to work in a Medicare approved facility with requisite policies and procedures in place. The UNE/Coledale Student-led Health and Wellbeing Clinic opened in March 2013.

### The 4G approach used

The approach used in the clinic was underpinned by the New England 4G Framework of Guided Self-Health developed by the Authors [41, 42] which is based on the United Kingdom National Health Service initiative Improving Access to Psychological Therapies [43] and expanded to include physical health conditions.

The 4G approach guided students to work in a collaborative investigate manner with individuals and families to tackle health and wellbeing issues. The first G was about ‘gathering information’ through comprehensive assessment and to pinpoint and prioritize problems. G2 was about ‘generating an agreed plan of action’ that included specific steps and timelines. G3 was ‘giving the person the educational and action based self-help materials’ specific to the work that they must undertake to address and tackle their health problem. The final G was the ‘guiding’ work the student or health practitioner undertook to keep the person or family on target to achieve their goal. This might involve brief follow up phone calls of five minutes to make sure people were staying connected to their plan. While much of this seems like an obvious action planning approach, a key strategy was engagement with the cognitive-behaviorally based self-help interventions that were readily and freely available. Get Self-Help is an example of where you might find materials to bespoke a treatment program [44].

### Requirement for record keeping

It was decided that accurate records needed to be kept to determine who was served, the issues involved and outcomes achieved. As part of a research study, residents were invited to keep their own health records including all on-site lab tests and physiological functioning results. It was discovered that residents preferred their records to be accessible but kept at the Centre. They also monitored their own progress using standardised measures including: Work and Social Functioning [45], the Patient Health Questionnaire [46], Generalized Anxiety [47], Problems and Goals [48], and measures specific to their health concerns. Students were trained to help residents to select and use evidence-based guided self-health packages. With increased health literacy, individuals in the community could make health decisions based on immediate feedback and support. For example, community members were encouraged to monitor their own blood glucose levels before and after exercise or meals or encouraged to study food labels and contents in order to make informed choices. Several examples below illustrate these outcomes.

## Results

In this section, we describe the overall achievements of the student-led clinic, offer three specific case studies as examples of the health outcomes achieved, and estimate the resultant cost savings to the health care system.

### Overall achievements

Since March 2013, students have been working with local service providers to establish the UNE/Coledale Student-led Health and Wellbeing Clinic. The Clinic received referrals from local GPs and other health agencies and self-referrals by members of the local community. Members of the teaching team included clinical facilitators, a nurse practitioner, a chronic disease clinical nurse consultant, mental health clinicians, and a range of other wellness professionals who work out of the Coledale Community Centre. Under the UNE banner of an integrated one-stop clinic, students were providing a wide range of evidence-based interventions—from triage, through assessment, testing, diagnosis, treatment, prescriptions, education and coaching.

Five founding students volunteered to undertake Coledale as a placement. The main reason for their participation was out of convenience to their home location. Students were instructed to apply outreach and engagement strategies. They began meeting and greeting community members as they came in for services. They liaised with health service agencies offering to do assessments and treatments within their scope of practice, under the supervision of the university project lead. Their mission was “to build health literacy and equity in a disadvantaged community while providing learning opportunities through a community governed student-led clinic that is person-centred, evolving, sustainable, and inter-professional”. In discussion with other community groups, the students recognised the need to “strengthen inter-professional partnerships across higher education, health and community sectors with private, government and non-government agencies” and adopted this as a goal.

The operation of the student-led clinic unfolded each day with a student selected team leader who identified the services and activities being held at the centre that day and consulted with agency staff on how students could assist. In pre-brief the students discussed, negotiated, assigned roles and recorded on paper a plan for the day. For example, “the wound nurse would like all clients pre-assessed as they arrive,” or “there is a men’s anger management group today and we are welcome to participate and offer health checks.” In addition to assisting agencies, students were assigned to community outreach and health promotion activities: “we need to develop a participation plan for the Homeless Connect event,” or “we need to work on our record keeping system.” Each day was different as determined by activities of the center and the number of residents dropping in for help which was usually around ten. The end of the day included a debrief of what happened, what was learned and what could be improved. Students confronted each other on their interpersonal dynamics with an action plan put in place.

Working one day per week, in the first three months, students assisted 278 community residents to obtain a free birth certificate and well over 100 residents to receive free drop-in help ranging from health checks, disease screening, treatments, prescriptions, education, health counselling, information about exercise, diet and health coaching, and emergency dental and medical referrals. As the demand for help increased, the clinic increased to three days per week. By the end of the first year, students had delivered over 1000 occasions of care, and a further 1500 local people were involved in health promotion initiatives. With the joining of an Aboriginal nurse practitioner as a student mentor and supervisor in early 2014, the clinic rapidly expanded into a five days per week operation. Although student placement hours, as attached to a unit of study was between 80 and 120 hours, a number of students continued to work beyond requirement in a volunteer capacity. Students rostered clinic coverage according to clinic activity needs and needs of students. So while some students contributed one day a week, others made a more intensive contribution. Flexible scheduling aimed to ensure maximum community engagement and continuity.

Students worked collaboratively with a wide range of health service providers including community nurses, local paediatricians, mental health practitioners and counsellors offering a variety of services including wound care, foot care, needle syringe program, renal transplant, antenatal, immunization and women’s health. Students also work with other community organisations such as those providing workplace development opportunities, cooking classes, church services, art classes, gardening groups, sport and fitness, breakfast clubs, after-school learning programs, learner driver education, men’s groups, and probation and parole. They were involved in, and have planned, special events such as health recognition days, and other programs to correspond to disease-specific awareness weeks.

Students have led and participated in a number of initiates such a re-write of the Indigenous Stroke Book, Birth Certificate Registration Day, Homeless Connect Day, Women’s Health Day, Hepatitis C and Sexual Health Day. More recently, they have taken on the eye and ear screening for all local elementary, middle and high schools. Ongoing groups such as Walk and Talk, Laughing Yoga, and Thai Chi have encouraged people from the neighborhood to join in health promotion activities. Increased community spirit and participation has been palpable as residents have access to a holistic approach to primary health care delivery that includes physical, occupational, social and emotional wellbeing. Within this mutually beneficial environment, reduced barriers to help and improved community relations are addressing health disparities.

### Case studies demonstrating health outcomes

Case records to date reveal that the student workforce has been able to detect life-threatening conditions and through their immediate action and referral, provide a positive reversal of conditions such as renal failure, heart attack, diabetic coma, bowel obstruction, head trauma, gangrene and fresh puncture wounds. A number of infectious and chronic diseases have been treated and monitored, including scabies, lice, impetigo, whooping cough, hypertension and diabetes. A few cases demonstrate the nature of student work. The scenarios described below are exemplars—cases that stand out because of their ability to capture the nature and essence of student and client collaboration in tacking health puzzles and problem solving that result in mutual benefit to student learning and positive client outcome. Students often referred to these cases when clinical or academic visitors came for a tour of the clinic because they depict the range of issues they dealt with. For a conference presentation, students drew on their notes in the clinical record to construct the following stories.

A man had been coming to the Community Centre bi-weekly for wound care from a community health nurse. When the student-led clinic started, students investigated why the wound was not healing. They discovered that the man had poor nutrition due to dental problems. Inaccessibility to dental care combined with a fear of needles kept this man from getting help. Students were able to spend time finding a dentist, working with the man’s needle phobia, arranging transport, and educating and supporting him to select nutritious foods which enabled his long standing wound to heal.

A young woman and her baby came to the clinic to escape a fight with her partner. A chat with the student uncovered domestic violence concerns. Students engaged the Anglicare Aboriginal counsellor and the Staying Home Leaving Violence coordinator who helped the woman consider her options. At the same time, a health check uncovered a punctured eardrum and an infection in the woman, and a skin disease on the baby. A pregnancy test was also found to be positive. Health teaching and prescriptions were delivered on the spot with ongoing follow up and counselling support.

An adolescent completing his community service at the Community Centre came in for a health check. While physically fit, the student asked the young man about any health changes he would like to make. By the end of the session, the two had been on the Internet downloading a “stop smoking” program. Over the weeks, the student coached the young man through the program and the goal to stop smoking was achieved. When asked about outcomes, the young man said that he felt good, had more money in his pocket and that his girlfriend said that he smelled better.

### Estimate of cost savings

The shortage of general practitioners who bulk bill in the community was calculated to leave 4000 residents without access to health care (personal communication, staff Medicare Local, 2013). With the arrival of the student-led clinic, residents sought help for a range of conditions that were often serious. From point of care testing, it was frequently found that residents were coping with known health problems but unaware of dangerous implications of treatment noncompliance or leaving problems unattended. Student triage records indicate that in addition to discovery of hidden yet severe problems, presentations ranged from stable conditions in need of urgent attention to minor injuries and chronic conditions requiring attention and ongoing monitoring. Again, because of the lack of GPs and the cost of those not bulk billing, the only available help for this population would have been the local hospital emergency department. It was estimated that the cost savings to the health care system were in the order of $437,000. This figure has been derived from two sources: 1) New South Wales Cost of Care Standards latest publication of 2009–2010 [49], and 2) personal communication with an administrator at the hospital nearest where the residents would be seek help (Coombs, 2013). As published, “the average cost of an emergency department presentation applicable in 2009–2010 was $396.00”. With an annual escalation factor of costs provided by the NSW Health Finance and Business Management Division to be an average 3.5 % per year, the cost of an emergency visit for 2013/2014 would have been approximately $450.00 [49]. This cost is based on a face-to-face consultation with minimal testing and intervention. Figures escalate exponentially when more extensive procedures are required.

The administrator reported the cost for the local hospital to be nearer to $490.00 per visit (personal communication with Coombs, 2013). Taking the lower figure of $450.00, with over 1000 occasions of care for individuals who would have had to seek help from an emergency department because of unavailability of GPs, the cost savings would be conservatively $450,000.

The average Medicare rebate cost of nurse practitioner services was $25.00 per visit. The nurse practitioner served near 500 clients with the help of students, which cost $12,500. Removing the Medicare rebate left a total savings of $437,500. With an additional 1500 other individuals receiving student-delivered health promotion and screening, it was difficult to determine costs savings from preventative care, screening, or early detection. That is the focus of a current study underway.

Other costs to consider include clinical facilitators who supervise students. These costs were not factored in because during this start-up period, the lead investigator of the grant supplied the supervision in consultation with other RNs working out of the Centre. However the actual cost of facilitation at an hourly rate for the first year of clinic development would have been $42,000. It should be noted however that the University is obliged to supply facilitators regardless of the location.

The figures reported here are from March 2013 to 2014—during the clinic start-up period with reduced operating hours. Since then, the students have served between 10 and 15 clients per day which, to date, converts to well over one million dollars of cost savings.

## Discussion

The main achievements of this particular student-led clinic were offering increased access to help in a disadvantaged community, offering the opportunity for collaborative learning for both students and community members and helping to renew the local workforce.

### Increased access to help

The individuals and families attending the clinic would not otherwise get help due to the extreme shortage of health practitioners, long waiting lists, transport and financial issues, and avoidance of services dues to stigma and previous negative experiences. Residents were drawn to students because of their accessibility, unlimited time to devote to each person, and willingness to learn together and from each other. Residents were empowered to explore their well-being issues with the removal of medical dominance. Increased community spirit and participation was palpable as residents had access to a holistic approach to primary health care delivery that included physical, occupational, social and emotional wellbeing. Within this mutually beneficial environment, reduced barriers to help and improved community relations the clinic aimed to make Coledale a healthier place for all. By improving access and health outcomes in an underserved population, this initiative also helped to reduce the overall burden of suffering and disease in a highly disadvantaged community.

### Collaborative learning

The UNE/Coledale student-led clinic was an invaluable and sustainable link between students, health care professionals, community based organizations, the university, and the community. The community benefited by learning to self-manage health and wellbeing issues. The students benefited by gaining practical experience in an interdisciplinary setting and through exposure to a community with unique and severe needs [50]. This model not only fulfilled the local, state, national and international health reform agenda but represented value for money. As a student training ground, services were delivered at no cost. Student and resident collaborative sharing and learning were beyond value.

### Renewal of the workforce

The HWA grant has offered a unique opportunity to think anew about how a range of vital community services can be offered more effectively and innovatively. Under the umbrella of the UNE community outreach plan and the UNE/Coledale Student-led Health and Wellness Clinic, an extensive range of community volunteers, elders, and agencies have joined to refresh the current workforce and prepared a developing workforce to enact state-of-the-art evidence-based help. This arrangement has enabled a common community engagement and service delivery plan that included sharing resources such as staffing (students, clinicians and practice managers) transport, databases, communication systems and helped to develop a shared research agenda.

## Conclusions

HWA awarded a substantial grant to the UNE School of Health (Nursing) to establish a student-led health clinic as a means to address the shortage of clinical training opportunities in rural and remote areas while, at the same time, providing a community service in priority health areas. Through this collaboration, local organisations were committed to strengthening inter-professional partnerships across higher education, health and community sectors with private, government and non-government agencies.

The student-led clinic has operated effectively within an underserved area of rural NSW. This model is worthy of consideration as it is strategically aligned with the agendas of the Australian Health Care Reform Alliance, Department of Health and Aging, and New South Wales Health. The outcomes were clearly an increased capacity to provide clinical experiences for students. With current health outcomes studies underway, it is projected that the clinic will: enable sustained positive health outcomes for residents; reduce the costs of disability and days lost to sickness; and decrease the impact of illness and suffering on persons, families, and communities. So far, routine data has supported these outcomes.

The student-led principles were the key to success and these were applicable to a wide range of situations. Students recognised the intrinsic value of all people and their right to high quality health care. As future professionals, they strove to understand the social determinants of health, the principles of primary care and the importance of socially responsive health promotion. Their vision was based on Coledale priorities, as determined through extensive communication with residents, community groups and health personnel. With this foundation, students created and maintained a student-led interdisciplinary approach to providing integrated and timely services to Coledale’s underserved population.

## Abbreviations

UNE: University of New England
NSW: New South Wales
HWA: Health Workforce Australia
ABS: Australian Bureau of Statistics
